# Systematic Review of Health Literacy and Health Behavior in Adolescents Research

**DOI:** 10.3390/epidemiologia7010029

**Published:** 2026-02-18

**Authors:** Saulius Sukys, Gerda Kuzmarskiene, Kristina Motiejunaite

**Affiliations:** Department of Physical and Social Education, Lithuanian Sports University, Sporto 6, LT-44221 Kaunas, Lithuania

**Keywords:** adolescents, health literacy, health behavior, systematic review

## Abstract

Background/Objectives: Despite the publication of several systematic reviews on adolescent health literacy, comprehensive evaluations of the relationship between health literacy and health-related behaviors are still limited. This systematic review sought to synthesize and critically appraise the available evidence on associations between health literacy and health behaviors among adolescents. Methods. A systematic search of three databases (Scopus, PubMed, and PsycINFO) was conducted in accordance with PRISMA guidelines. Thirty-seven eligible cross-sectional studies were selected for qualitative synthesis. Methodological quality was evaluated using the Newcastle–Ottawa Scale adapted for cross-sectional studies. Results: The 37 included studies encompassed 71,558 adolescents (mean age range 11.0–17.0 years) and were conducted primarily in Europe (n = 22), with additional studies from the USA (n = 5), Asia (n = 8), and cross-cultural settings (n = 2). Across studies, 11 HL instruments were used (including two eHealth literacy measures), most commonly the Health Literacy for School-aged Children scale (n = 14). Physical activity (n = 22), nutrition-related indicators (n = 26), and smoking/alcohol/drug outcomes (n = 16) were assessed using heterogeneous operationalisations. Overall, higher HL was more often associated with healthier behavioral profiles, with more consistent patterns for nutrition-related outcomes. Findings for physical activity and substance use were more heterogeneous and, in some cases, varied depending on the HL measurement approach (subjective vs. objective) and the behavioral reference period. Conclusions: Current evidence indicates that higher health literacy in adolescents is generally associated with more favorable health behaviors, particularly regarding nutrition-related indicators. However, study heterogeneity and the predominance of cross-sectional designs limit comparability and causal inference. Future research should prioritize standardized measurement tools and longitudinal designs to clarify directionality and underlying mechanisms.

## 1. Introduction

Adolescence is a pivotal stage of a critical developmental period when many important health-related behaviors are formed, such as physical activity, dietary practices, and substance use, and are established and may carry forward into later life. Evidence suggests that physical activity shows low-to-moderate stability over time, including continuity from adolescence into adulthood, supporting the importance of early behavioral patterns for long-term health trajectories [1]. Similarly, dietary behaviors are shaped during late adolescence and the transition into early adulthood, a period characterized by substantial lifestyle changes and shifting social roles that may influence food-related choices and routines [2]. Health-related behaviors also tend to co-occur rather than emerge in isolation; for example, longitudinal evidence indicates strong links between smoking and alcohol use and inverse associations between smoking and physical activity, alongside marked continuity of smoking over time [3]. These behavioral patterns are embedded in social contexts and shaped by family resources and parenting, with school and peer influences becoming increasingly salient as young people grow [4]. At the same time, adolescents navigate an expanding digital information environment in which social media use and exposure are linked to health-relevant outcomes, and where persuasive content may be amplified through social feedback dynamics [5]. In addition, social media influencers can function as salient role models and sources of health-related content; however, influencer content may be shaped by commercial interests, vary in health expertise, and include embedded promotion of unhealthy products (e.g., energy-dense foods, tobacco-related products, and alcohol), raising concerns about misinformation and persuasive marketing [6]. Given adolescents’ frequent exposure to health-related content in online environments—where information quality, persuasive intent, and commercial influences vary considerably, and misinformation can circulate rapidly—the capacity to critically assess credibility and to apply information effectively can be conceptualized as a key upstream determinant of health-related behavioral decisions [7].

Health literacy (HL) is broadly defined as “people’s knowledge, motivation and competences to access, understand, appraise, and apply health information in order to make judgments and take decisions in everyday life concerning healthcare, disease prevention and health promotion to maintain or improve quality of life during the life course” [8]. This conceptualization highlights not only functional skills but also higher-order competencies (e.g., critical appraisal and the application of information) that may be particularly relevant to adolescents’ behavioral choices in complex informational contexts.

Despite the well-documented benefits of HL across diverse populations, research synthesizing findings specific to adolescents remains limited yet critically important. Several systematic reviews have sought to address this gap, each emphasizing different dimensions of adolescent HL. Some studies have assessed the effectiveness of interventions designed to improve student’s HL [9,10,11] including school-based HL interventions aimed socioeconomically disadvantaged adolescents [12]. Others have concentrated on the developing and validating instruments to measure HL in children and adolescents [13].

In addition, region-specific analyses have provided valuable contextual insights. For instance, Choudhry et al. [14] conducted a scoping review of student HL in Australia, Sarhan et al. [15] examined studies from the eastern Mediterranean region, Olyani and Peyman [16] examined the relationship between HL and health behavior among Iranian adolescents, and Mao et al. [17] analyzed HL research among Chinese students and its associated determinants. Beyond regional focus, other reviews have explored specific health outcomes, such as the relationship between HL and musculoskeletal disorders in adolescents [18] and the associations between HL and health behaviors among pediatric populations [19]. Navarro Rubio and Blay [20] conducted a review of systematic reviews of HL in children and adolescents. Collectively, these reviews demonstrate growing interest in adolescent HL while also indicating that evidence on its links with concrete health outcomes and behaviors remains fragmented.

Nevertheless, broad, integrative syntheses that systematically examine the overall relationship between adolescent HL and a wide range of health-related behavior indicators are still limited. A notable exception is the systematic review by Fleary et al. [21], which synthesized existing evidence on associations between HL and health behaviors in adolescents. While this review provided important insights into the health literacy–health behavior nexus, the accumulation of new empirical studies since its publication highlights the need for an updated and more comprehensive synthesis. Accordingly, the present review aims to consolidate and critically analyze current research examining the relationship between HL and health behaviors in adolescents.

## 2. Materials and Methods

This review was conducted in accordance with the PRISMA 2020 guidelines (Supplemental Table S1) [22]. The protocol of this analysis was registered with PROSPERO at the following link: https://www.crd.york.ac.uk/PROSPERO/view/CRD420251186167 (accessed on 7 November 2025).

### 2.1. Search Strategies

In accordance with PRISMA guidance, we conducted a comprehensive search of three international electronic databases (SCOPUS, PubMed, and PsycINFO) to identify relevant studies. The search strategy was structured around the following three concept blocks: (1) population (adolescents), (2) exposure (HL), and (3) outcomes (health behaviors and related outcomes). Within each concept block, synonymous terms were combined using the Boolean operator OR, and the three concept blocks were combined using AND. Database-specific field tags were applied (e.g., Title/Abstract in PubMed; TITLE-ABS-KEY in Scopus; and Title/Abstract/Keywords in PsycINFO), and truncation/wildcards (e.g., adolescen*, behavio*r) were used where appropriate to capture variant word endings and spelling. An example of the full search strategy is provided in the Supplementary Table S1.

The search strategy was piloted in January 2024 and updated in February 2024. Searches were limited to records published between January 2019 and December 2024.

### 2.2. Eligibility Criteria

For this systematic review, only articles published in English were included. As our study’s primary focus was on adolescents aged 10–19 [23], we set cut-off points by excluding studies with participants aged over 19, a mean over 19, or a mean lower than 10. If the age range fell outside the criterion, articles were included only if results were presented for subgroups (e.g., 15–19 years from a sample aged 15–40). We excluded studies that measured non-general or digital HL or in which HL was a dependent variable. We excluded studies without statistical analyses of the relationship between HL and health behavior. Detailed inclusion and exclusion criteria are presented in Table 1.

### 2.3. Study Selection

Results from the database searches were imported into RefWorks. The search yielded 3773 articles. Duplicated articles (n = 1306) were removed, and 2467 studies remained for title and abstract screening. Two reviewers independently screened all titles and abstracts against the predefined eligibility criteria. Disagreements were resolved through discussion among the author team. The percentage of agreement of our screening process was 94.23%, which is an acceptable level [24]. After discussion, the authors reached a final decision on all discrepancies. Full-text analysis (including additional references) was also conducted independently by two authors. The percentage agreement from our full-text screening was high (94.65%). After resolving discrepancies through discussion of all authors, a total of 37 articles were considered eligible for inclusion in the systematic review (list of included articles, Supplemental Table S3).

### 2.4. Data Extraction and Risk of Bias Assessment

Data were extracted using a pre-piloted, standardized data extraction form developed specifically for this review. Two reviewers independently extracted data from each included study. The following information was collected: (i) bibliographic details (first author, year), country/study setting, and study design; (ii) participant characteristics (sample size, age range/mean age, and sex distribution); (iii) the HL construct and its measurement (name of the instrument, scoring procedure, and applied cut-off criteria, where available); (iv) definitions and measurement methods of the outcomes; and (v) quantitative results describing the association between HL and the outcomes (e.g., correlation coefficients, regression coefficients, odds ratios or relative risks, and group differences), together with precision estimates (95% confidence intervals, standard errors) and p values, where reported. When multiple statistical models were presented, priority was given to the most fully adjusted estimates, and the list of covariates included in these models was also recorded. Any uncertainties or missing information were clarified by consulting full-text articles and Supplementary Materials. Any disagreements between reviewers were settled through discussion; when consensus was not achieved, a third reviewer made the final decision.

The risk of bias of the included observational studies was assessed independently by two reviewers [25] using the Newcastle–Ottawa Scale (NOS) [26] adapted for cross-sectional studies [25]. This tool evaluates potential bias in domains related to participant selection, comparability of study groups (to control for confounding), and outcome assessment. A total NOS score was assigned to each study, and overall methodological quality was predefined and categorized as low (0–5 points), moderate (6–7 points), or high (8–10 points). Any disagreements in scoring were resolved through discussion and, when necessary, by involving a third reviewer. The risk of bias assessments was incorporated into the interpretation of the findings and used to contextualize the strength of the evidence across different outcome domains (risk of bias assessment for included studies, Supplemental Table S4).

## 3. Results

**Figure 1:**
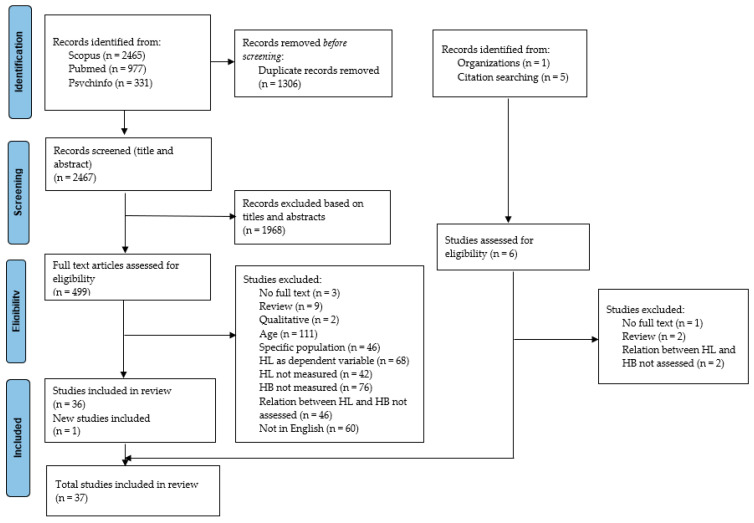
The PRISMA flowchart of the study selection process.

Among 2467 nonduplicate studies identified, 1968 were excluded based on titles and abstracts. The remaining 499 studies underwent full-text assessment for eligibility. Ultimately, 36 studies reporting association between HL and health-related behaviors were included in the qualitative analysis. Additionally, five studies were evaluated for eligibility, and one of them was included in the final analysis. The detailed study selection process, with reasons for exclusion at the screening steps, is shown in Figure 1.

### 3.1. Study Characteristics and Populations

From all studies included in this analyses 22 studies were conducted in Europe (Turkey n = 9, Austria n = 1, Italy n = 1, Poland n = 3, Slovakia n= 1, Greece n = 1, Croatia n = 1, Finland n = 2, Lithuanian n = 3), five in the United States of America, eight in Asia (China n = 2, Indonesia n = 1, Thailand n = 1, Iran n = 3), and two cross-cultural studies (Table 2).

In total, 71,558 adolescents participated in all selected studies. The study population varies, with adolescents having a mean age ranging from 11.0 [27] to 17.0 [28,29]. The number of participants in the study ranged from 167 [30] to 22,628 [31].

All studies were cross-sectional in design. Of the 38 studies, 35 measured general HL and three eHealth literacy. The health behaviors investigated were physical activity (n = 2) [32,33], nutrition and dietary related behavior (n = 8) [28,34,35,36,37,38,39,40] alcohol drinking, smoking and substance use (n = 6) [29,41,42,43,44,45], both physical activity and nutrition (n = 11) [27,30,46,47,48,49,50,51,52,53,54], both physical activity and alcohol drinking (n = 2) [31,55], and range of health behaviors, i.e., physical activity, nutrition, alcohol drinking, and smoking (n = 8) [56,57,58,59,60,61,62,63].

### 3.2. Assessment of Health Literacy

The measures used in the studies are presented in Table 2. Eleven unique measures of HL were used across selected studies (two of them measure eHealth literacy). Most used research tools subjectively measure HL. Among these tools the HL for School-aged Children [64] was used in 14 studies [33,34,37,39,44,45,46,47,48,50,51,53,61,62], assesses children’s HL as a broad set of knowledge and competencies that people seek to encompass, evaluate, construct, and use the theoretical knowledge, practical knowledge, critical thinking, self-awareness and citizenship [64]. The European Health Literacy Survey Questionnaire short (HLS-EU-Q16) and long (HLS-EU-47, [65]) forms were used in five studies [28,29,32,42,63]. These questionnaires ask participants to rate their perceived difficulty in finding, understanding, appraising, and applying health information in the context of health care, disease prevention, and health promotion. The Health Literacy Measure for Adolescents (HELMAs, [66]) was used in three studies [40,41,60], which measure the following eight HL dimensions: access, reading, understanding, appraisal, use, communication, self-efficacy, and numeracy. The Health Literacy Assessment Tool (HLAT-8, [67]) was used in two studies [58,59] to measure the ability to access, understand, evaluate, and communicate health information in the context of family and friends. In one study by Yang et al. [31], the Chinese Adolescent Interactive Health Literacy Questionnaire (CAIHLQ, [68]) was used, which measures six dimensions (physical activity, interpersonal relationships, stress management, self-actualization, health awareness, and dietary behavior) of HL. Also, in one study, a single index scale measuring participants’ knowledge of several lifestyle and nutritional factors affecting health status [27]. The Short Health Literacy (HLS19-Q12, [69]) was used in one study [55]. In seven studies, HL was measured by objective measures. On of these measures was the Newest Vital Sign (NVS, [70]) to assess functional HL and was used in five studies [30,36,38,54,57,58] and another—was the Assessments of Adolescent Health Literacy (AAHL, [71]) to measure functional, interactive and critical HL was used in two studies [43,56]. In three studies, digital HL was measured, and one of them was the e-Health Literacy Scale (eHEALS, [72]) [49,52], and another—was Media Health Literacy (MHL, [73]) [35].

**Table 2:** Characteristics of the included studies.

Author (Year)	Country	Study Population Characteristics	HL Measure	Assessed Health Behavior	Statistical Analysis	Main Results	Risk of Bias
Ayaz-Alkaya et al., 2021 [46]	Turkey	N = 810; Age range: not specified; Mean age = 12.98 years.	Turkish version of HLSAC	Physical activity, nutrition	Descriptive statistics, *t*-test, ANOVA, Pearson correlation	Positive correlation was observed between HL and healthy nutrition–exercise behavior (r = 0.345 ***) as well as meal pattern (r = 0.230 ***).	Moderate
Ayaz-Alkaya et al., 2024 [47]	Turkey	N = 1046; Age range: 11–14 years; Mean age = 12.42 years.	Turkish version of HLSAC	Physical activity, nutrition	Descriptive statistics, *t*-test, ANOVA, multiple linear regression	HL positively correlated with health promotion behaviors (β = 0.489 ***).	High
Azarang et al., 2024 [41]	Iran	N = 275; Age range: 15–18 years; Mean age = 16.86 years.	HELMA	Substance use	Pearson correlation, *t*-test, ANOVA, multiple linear regression	Significant negative correlation between HL and addiction susceptibility (r = –0.66 ***).	High
Bektas et al., 2021 [48]	Turkey	N = 440; Age range: 13–18 years; Mean age = 15.22 years.	Turkish version of HLSAC	Physical activity, nutrition	Descriptive statistics, Pearson correlation, multiple linear regression	HL levels significantly predicted the nutrition sub-dimensions of the healthy lifestyle behavior of the adolescents (β = 0.177 *).	High
Brandt et al., 2019 [42]	Austria	N = 5614; Age range: 13–17 years; Mean age not specified.	Adapted version of HLS-EU-Q16	Tobacco, alcohol use	Structural Equation Modeling (SEM), Confirmatory Factor Analysis (CFA), correlation and regression analyses	Lower HL was associated with higher cigarette smoking both over the lifetime (β = 0.12 ***) and in the past 30 days (β = 0.15 ***). Similarly, lower HL was linked to more frequent alcohol use (lifetime: β = 0.03 *; last 30 days: β = 0.07 ***).	Moderate
Delbosq et al., 2022 [34]	Italy	N = 2145; Age range: 13–15 years; Mean age not specified.	Italian version of HLSAC	Fruit, vegetable, sweets, and soft drink consumption; breakfast frequency	Multiple binary logistic regressions	HL was significant predictor of daily consumption of fruit (B = 0.026 *), vegetable (B = 0.244 ***), not related with sweet consumption, significantly negatively related with soft drinks consumption (B = −0.024 *). Not related with breakfast consumption frequency.	High
Duplaga et al., 2021 [28]	Poland	N = 2223; Age range: not specified; Mean age = 17.01 years.	Adapted version of HLS-EU-Q47	Number of meals/day, meal regularity, largest meal, fruit/vegetable and fast-food consumption	Univariate and multivariate logistic regression	Participants with higher HL were more likely than those with lower HL to consume fruit and vegetables at least once per day (OR = 1.03 ** (95% CI: 1.01–1.04). HL was a significant predictor (OR = 0.98 * (95% CI: 0.95–0.999) for fast food consumption.	High
Duplaga et al., 2022 [29]	Poland	N = 2223; Age range: not specified; Mean age = 17.1 years.	Adapted version of HLS-EU-Q47	Tobacco use	Univariate and multivariate logistic regression	HL not significantly related with using cigarettes ever in past and in the last month.	High
Fleary et al., 2023 [56]	USA	N = 380; Age rage: 12–19 years; Mean age = 15.98 years.	AAHL	Physical activity, fruit and vegetable, sugar-sweetened drinks, junk food, smoking, vaping, alcohol, binge drinking	Pearson correlations, hierarchical regressions, moderation	Functional HL negatively related with use of sugar-sweetened beverage (β = −0.13 *) and smoking cigarettes during past 30 days (β = −0.16 *). Critical HL negatively related with use of sugar-sweetened beverage (β = −0.24 **). Interactive/communicative HL positively related with daily physical activity (β = 0.11 *) and negatively with alcohol use (β = −0.19 *).	High
Fleary & Joseph, 2024 [57]	USA	N = 300; Age range: 13–17 years; Mean age not specified.	NVS	Physical activity, fruit and vegetable, sugar-sweetened drinks, junk food, smoking, vaping, alcohol, binge drinking	Pearson correlations; Actor–Partner Interdependence Model	Adolescents’ HL showed negative correlations with sugar sweetened beverages consumption (*p* < 0.05), sedentary activity (*p* < 0.01), cigarette smoking (*p* < 0.01), vaping (*p* < 0.01), and binge-drinking (*p* < 0.01).	High
Fleary et al., 2024 [43]	USA	N = 675; Age range: 13–18 years; Mean age = 15.5 years.	AAHL	Substance use avoidance (alcohol, vaping, and cigarette smoking)	Binary and multinomial logistic regression, hierarchical logistic regressions	Higher interactive (OR = 2.12 * (95% CI: 1.12–4.00) and composite HL (OR = 2.06 * (95% CI: 1.10–3.84) were significantly associated with substance use avoidance.	High
Guo et al., 2020 [58]	China and Australia	N = 770; Age range: 11–17 years; Mean age = 13.45 years.	HLAT-8; NVS, HLS-47	Physical activity, breakfast eating, smoking, alcohol.	Descriptive, *t*-test, ANOVA, linear and logistic regression	HL was positively associated with health-promoting behaviors when using the HLAT-8 (β = 0.06 *) and the HLS-47 (β = 0.07 *), but no significant relationship when using the NVS.	High
Guo et al., 2021 [59]	China	N = 650; Age range: 11–17 years; Mean age = 13.42 years.	Chinese version of HLAT-8	Physical activity, breakfast eating, smoking, alcohol.	Descriptive, *t*-tests, ANOVA, Pearson/Spearman correlations; Path analysis (SEM)s	Significant and direct relationship between HL and physical activity (r = 0.14 *), but not significant relationship with breakfast eating, cigarette smoking, and alcohol drinking.	High
Gürkan, & Ayar, 2020 [49]	Turkey	N = 219; Age range: 14–18 years; Mean age = 16.52 years.	Turkish version of the eHEALS	Physical activity, nutrition behavior	Descriptive statistics, Pearson correlation, simple linear regression	Significant positive association between e-HL and health promotion behaviors (β = 0.416 ***). e-HL positively associated with nutritional behavior subscale (β = 0.270 *) and exercise subscale (β = 0.122 *).	Moderate
Hnidková et al., 2024 [50]	Slovakia	N = 508; Age range: 14–15 years; Mean age = 14.50 years.	Slovak version of the HLSAC	Physical activity, body composition	Linear regression, mediation analysis	Higher HL was directly associated with higher moderate-to-vigorous (β = 0.005 ***) and vigorous physical activity (β = −0.08 ***), HL indirectly influenced body composition through physical activity. No direct association with BMI.	High
Huang et al., 2024 [60]	Hong Kong	N = 777; Age range: not specified; Mean age = 13.57.	Chinese version of the HELMA	Physical activity, diet (fruits, vegetables, breakfast), smoking, alcohol use	Multivariate logistic regression	Desirable HL was negatively associated with insufficient intakes of vegetables (OR = 0.43 *** (95% CI: 0.28–0.67) and fruits (OR =0.58 ** (95% CI: 0.4–1–0.81), skipping breakfast (OR = 0.64 ** (95% CI: 0.45–0.91), physical inactivity (OR = 0.56 * (95% CI: 0.35–0.90). Not related with smoking and alcohol drinking.	High
Jindarattanaporn et al., 2023 [35]	Thailand	N = 1871; Age range: 10–14 years; Mean age = 11.9 years.	MHL	Fruit and vegetable consumption	ANOVA, *t*-test, multiple linear regression	Adolescents with higher MHL were more likely to consume fruit (β = 0.085 ***) and vegetables (β = 0.101 ***) more often.	High
Kanellopoulou et al., 2022 [27]	Greece	N = 1728; Age range: 10–12 years; Median age = 11 years.	Item Response Theory-based composite index	Physical activity, dietary habits (Mediterranean diet adherence, breakfast, meals/day, eating out frequency), body composition	Descriptive stats, χ^2^, *t*-test; multiple linear regression	Children with higher l HL were more likely to eat breakfast daily (*p* < 0.001), and to have more than three meals per day (*p* < 0.001). They also ate out less often (*p* < 0.01), and ordered takeaway food less frequently (*p* < 0.001). In addition, they more regularly had a homemade brunch (*p* < 0.001), rather than buying one from the school canteen (*p* < 0.001), and they more often eat meals together with their whole family (*p* < 0.001).	High
Karagözoğlu & İlhan, 2024 [51]	Turkey	N = 649; Age range: 14–18 years; Mean age = 15.54 years.	Turkish version of HLSAC	Physical activity, nutrition	Descriptive stats; *t*-test, ANOVA, multiple regression	HL significantly positively predicted physical activity (β = 0.163 ***) and nutrition (β = 0.180 ***).	High
Kesic et al., 2022 [32]	Croatia	N = 247; Age range: not specified; Mean age = 16.8.	HLS-EU-Q47	Physical activity	Correlations, K-means clusters, ANOVA	HL was not significantly associated with physical activity.	High
Kinnunen et al., 2022 [44]	Finland, Germany, Netherlands	N= 5088; Age range: 12–19 years; Mean age = 14.65 years.	HLSAC	Substance (smoking, alcohol, cannabis) use	Logistic regression, GLMM, path analysis	Lower HL significantly associated with weekly smoking (OR = 2.32 *** (95% CI: 1.56–3.45), monthly alcohol use smoking (OR = 2.32 *** (95% CI: 1.56–3.45).	High
Kleszczewska et al., 2022 [45]	Poland	N= 1663; Age range: not specified; Mean age = 17.63 years.	HLSAC	Risk behavior index (smoking, alcohol, marijuana use)	Manan Whitney, Kruskal–Wallis tests, ANOVA, linear regression	Higher HL significantly related with lower Risk Behavior Index (β = −0.063 **).	High
Korkmaz Aslan et al., 2021 [52]	Turkey	N = 409; Age range: 14–19 years; Mean age = 16.0 years.	eHEALS	Nutrition, exercise	Multiple regression analysis	e-HL significantly and positively predicted nutrition (β = 0.64 ***) exercise (β = 0.36 ***).	High
McCormick et al., 2021 [36]	USA	N = 793; Age range: 11–13; Mean age = 12 years.	NVS	Sugar-sweetened beverage intake	Descriptive statistics; ANOVA; stepwise regression analysis	Higher sugar-sweetened beverage intake was associated with lower HL, less favorable behavioral intentions, affective attitudes, and perceived behavioral control.	High
Motemedi et al., 2020 [38]	Iran	Cross-sectional; N = 439; Age range: 15–18 years; Mean age = 16.5 years.	The Newest Vital Sign (NVS)	Body composition	Descriptive statistics; *t*-tests; ANOVA; Spearman correlation	Students with higher HL had significantly healthier BMI profiles (*p* < 0.0001).	Moderate
Ozturk Eyimaya & Tezel, 2024 [61]	Turkey	N = 1228; Age range: 10–15 years; Mean age = 11.7 years.	Turkish version of HLSAC	Exercising, nutrition, smoking	*t*-tests, ANOVA, linear regression	Weak negative linear relation between HL and nutrition (r = −0.083 **), exercise (r = −0.238 ***), smoking (r = −0.088 **).	High
Ozturk Haney, 2020 [37]	Turkey	N = 204; Age range: 11–14 years; Mean age = 12.8 years.	Turkish version of HLSAC	Body composition	Descriptive statistics, χ^2^, *t*-test, ANOVA, multiple regression analysis	No significant correlation between child HL and BMI (r = 0.04).	High
Ozturk & Ayaz-Alkaya, 2020 [53]	Turkey	N = 2498; Age range: 11–15 years; Mean age = (mean 11.7 ± 1.2).	Turkish version of HLSAC	Nutrition, exercise	Descriptive stats, *t*-tests, ANOVA, Spearman correlation	HL positively linearly related with nutrition (r = 0.282 ***) and exercise (r = 0.247 ***).	High
Paakkari et al., 2019 [62]	Finland	N = 3833; Age range: 13–15 years.	HLSAC	Physical activity, healthy food, smoking, alcohol use	Pearson correlations, path analysis	HL positively related with physical activity (β = 0.17 ***), use of healthy foods (β = 0.20 ***), and negatively with smoking (β = −0.08 ***) and alcohol use (β = −0.12 ***).	High
Prihanto et al., 2021 [63]	Indonesia	Cross-sectional; N = 960; Age range: 14–19 years; Mean age = 16.2 years.	HLS-EU-Q16	Physical activity, smoking, alcohol use, drug use	Descriptive stats, χ^2^ tests, binomial logistic regression	Comprehensive HL positively predicted physical activity (OR = 1.8 (95% CI: 0.8–3.8) and not use drug (OR = 9.3 * (95% CI: 2.1–41.3). Functional HL contributed to no smoking behavior (OR = 6.8 *** (95% CI: 2.9–15.9) and alcohol use (OR = 2.2 ** (95% CI: 1.3–3.9).	High
Puupponen et al., 2021 [39]	Finland	Cross-sectional; N = 7405; Age range: 13 and 15 years.	HLSAC	Energy drink consumption	Descriptive stats; multilevel mixed-effects binary logistic regression	Lower HL significantly associated with higher weekly energy drink consumption among 13-year-olds (OR = 1.75 ** (95% CI: 1.22–2.49) and 15-years-olds (OR = 1.52 * (95% CI: 1.07–2.16).	High
Reid et al., 2021 [54]	USA	Cross-sectional; N = 854; Age range: 11 and older; Mean age = 12.0 years.	NVS	Energy-balance-related behaviors: water, fruit/veg intake, junk food, sugar-sweetened beverages, physical activity	Nonparametric Hodges–Lehmann median difference test	Adolescents with limited HL had significantly lower fruit/vegetable intake, physical activity, and higher sugar-sweetened beverages, junk food, screen time and BMI.	Moderate
Sukys et al., 2024 [55]	Lithuania	Cross-sectional; N = 809; Age range: 15–19 years; Mean age = 16.4 years.	Lithuanian version of HLS19-Q12	Physical activity, smoking, alcohol use	EFA + CFA, *t*-tests, ANOVA, χ^2^, Pearson r, multiple linear regression	Higher HL positively related to physical activity (β = 0.10 **), negatively to lifetime smoking (β = −0.10 **) and alcohol use (lifetime β = −0.14 ***; past 30 days β = −0.09 **).	High
Sukys et al., 2021 [33]	Lithuania	Cross-sectional; N = 2369; Age range: 13–16 years; Mean age = 14.5 years.	Lithuanian version of HLSAC	Physical activity	χ^2^ tests and binary logistic regression	Moderate and high HL levels were significantly positively associated with being physically active during leisure time (OR = 0.56 *** (95% CI: 0.45–0.70)).	High
Rutkauskaite & Kuusinen, 2019 [30]	Lithuania	Cross-sectional; N = 167; Age range: 14–18 years; Mean age = 16 years.	NVS	Physical activity, body composition	Nonparametric tests: spearman r, Mann–Whitney, Kruskal–Wallis, χ^2^	No significant associations between HL and physical activity or BMI.	Moderate
Yang et al., 2019 [31]	China	N = 22,628; Age range: not specified; Mean age = 15.36.	CAIHLQ	Smoking, alcohol consumption	Multinomial logistical regression	Higher HL associated with lower probability of smoking/alcohol use and screen time (OR = 0.990 ** (0.982–0.998)).	High
Zare-Zardiny et al., 2021 [40]	Iran	Cross-sectional; N = 423; Age range: 15–19 years; Mean age = 16.8 years.	HELMA	Body composition	Descriptive stats, Pearson correlation, *t*-tests, ANOVA, multiple regression	HL do not relate to BMI (β = −0.03).	High

Note: HL: health literacy, *HLSAC:* Health Literacy for School-aged Children Scale, *HELMA:* Health Literacy Measure for Adolescents, *HLS-EU-Q16:* European Health Literacy Survey Questionnaire, *HLS-EU-Q47:* European Health Literacy Survey Questionnaire, *AAHL:* Assessments of Adolescent Health Literacy, *NVS:* The Newest Vital Sign, *HLS-47*: 47-item Health Literacy Study-Asia-Questionnaire, *HLAT-8:* the eight-item Health Literacy Assessment Tool, *eHEALS:* e-Health Literacy, *CAIHLQ*: The Chinese Adolescent Interactive Health Literacy Questionnaire, *MHL:* Media Health Literacy. BMI: body mass index. * *p* < 0.5, ** *p* < 0.001, *** *p* < 0.001.

### 3.3. Relationship Between Health Literacy and Physical Activity

Among all the studies included in this analysis, 22 assessed the relationship between HL and physical activity. Most of these studies were conducted in Europe (n = 15), and adolescents’ HL was measured by subjective general HL measures. In just one study [30], an objective measure was used. Three studies were conducted in the USA and measured HL by objective instruments. Four studies were conducted in Asia and Australia, and among these studies, one measured HL by subjective and objective measures [58]. The most common measure of physical activity was self-reported number of days per week in which adolescents were physically active for at least 60 min per day [30,32,33,50,54,55,56,57,58,59,60,62,63]. In four studies, physical activity was used as an indicator of the health promotion behavior scale [47,49,52,53]. In three studies, physical activity was one of the adolescents’ lifestyle profile indicators [46,48,51]. In one study, physical activity was a subdimension of the Cardiovascular Health Behavior Scale for Children and was measured by four-item scales with response options from always to never [61].

In all studies conducted outside Europe, findings indicated a positive relationship between general HL and physical activity. Two studies in Europe [30,32] found no association between HL and physical activity. Only in two studies was the relationship of physical activity with digital HL assessed [49,52], and a positive association was found. One study found that the relationship between HL and physical activity depends on the HL measure [58]. Significant relationships were found when measuring HL with subjective measures, and no relationship was found when HL was measured with objective instruments. It is also important to note that several studies reporting positive associations between HL and physical activity did not adjust for key sociodemographic or family-level confounders and received low scores (including zero points) in the comparability domain, which reduces confidence in the robustness of these associations.

### 3.4. Relationship Between Health Literacy and Smoking, Alcohol and Drug Use

Among all studies, 16 assessed the relationship between HL and smoking, alcohol drinking, and drug use. Seven of these studies were conducted in Europe, three in the USA, and six in Asia and Australia. In two studies, just smoking was measured [29,61]. In three studies, the association between HL and drug (marijuana, cannabis, amphetamines, or methamphetamines) use [44,45,63] and one association with positive thoughts towards drugs [41] was measured.

In one study, alcohol use was measured in the last 12 months [44]; in two studies, smoking and alcohol use were measured in the last 30 days and in lifetime [42,55]. In most studies, smoking and alcohol use were measured just in a 30-day period [31,44,45,56,57,58,59,60,63]. In one study, e-cigarette smoking was measured in the last month or ever used [29]. In one study, participants had to report whether they had ever tried alcohol, used an electronic vapor product, and/or tried cigarette smoking, even one or two puffs [43]. In one study, adolescents had to report how often they currently smoke or drink alcohol [62]. In one study, smoking was a subdimension of the Cardiovascular Health Behavior Scale for Children [61]. Drug use in three studies was measured in the period of the last 30 days [45], in one during the last 12 months [44], and lifetime [63].

In most studies, negative relationships between HL and smoking or alcohol use in the past 30 days [31,42,44,45,56,57,62,63]. However, some published studies did not identify an association between HL and smoking or alcohol use in the past 30 days was found [55,59], although a significant negative association was observed when smoking and alcohol use were assessed as lifetime behaviors [55]. No association was found between HL and smoking when no specific reference period was defined [61]. One study reported divergent patterns depending on the HL instrument used [58]: a significant association with smoking and alcohol use was detected when HL was measured with HLAT-8, whereas no significant associations were observed when it was assessed using the NVS. Kinnunen et al. [44] study included adolescents from three different European cities. When the pooled data were analyzed, an association was found between HL and smoking and alcohol use, but city-specific analyses did not reveal significant associations in all settings. With regard to illicit drug use, a study conducted in Indonesia showed that sufficient HL was associated with no drug abuse [63], whereas in Europe, one study found no significant associations between HL and cannabis use [44]. However, another European study found significant negative associations between HL and an adolescent risk behavior index, in which marijuana use was one of the indicators [45]. One study in Iran reported a negative correlation between HL and adolescents’ positive thoughts towards drugs [41].

### 3.5. Relationship Between Health Literacy and Nutrition

Among all the studies included in this analysis, 26 assessed the relationship between HL and nutrition-related indicators. Fifteen of these studies were conducted in Europe, four in the USA, and seven in Asia and Australia. Nutrition was measured as fruit and vegetable, sugar-sweetened beverage, and junk food use [28,34,54,56,57]. Other studies focused just on vegetable, fruit, and also breakfast eating [60], as others just on breakfast eating [58,59] or just on fruit and vegetable eating [35,62], or just on sugar-sweetened products use [36,39]. In three studies, nutrition was one of the adolescents’ lifestyle profile indicators [46,48,51]. In four studies, nutrition was used as an indicator of the health promotion behavior scale [47,49,52,53]. One study examined adherence to the Mediterranean diet, breakfast consumption, and eating meals at home or takeaway [27]. In one study, nutrition was a subdimension of the Cardiovascular Health Behavior Scale for Children [61]. In 12 studies, data on body composition or weight perception were also measured [27,30,34,35,37,38,40,46,47,50,51,54].

Six studies found that HL was a significant predictor of daily fruit and vegetable consumption [28,34,35,54,60,62]. However, not all of these studies achieved high scores in the comparability domain, and only a subset adjusted for core confounders such as socioeconomic status or parental education. Associations observed in better-adjusted models are therefore more informative for drawing inferences than those based on unadjusted or minimally adjusted analyses. In one study, only critical HL was associated with fruit and vegetable use [56], whereas in another study, no significant relationship was reported between HL and fruit and vegetable use [57]. Four studies found that adolescents with limited HL had significantly higher consumption of sugar-sweetened beverages and junk food [36,54,57]. One study reported significant relationships between critical HL and sugar-sweetened beverage intake [56]. One study separately analyzed media HL and did not find a relationship with sugar-sweetened beverages [36]. Two studies found that children with higher HL consumed breakfast every day [27,60], whereas two did not find a relationship between HL and breakfast consumption frequency [34,59]. In one study, HL was measured using three different measures (including eating breakfast as one indicator of health behaviors) and yielded different results. HL was positively associated with health-promoting behaviors when using the HLAT-8 and the HLS-47, but related when using the NVS [58]. One study also reported that higher HL is positively related to eating at home with the whole family [27]. Across all studies that used a healthy lifestyle profile, HL levels significantly predicted the nutrition sub-dimension [46,48,51]. Also, in all four studies that used the health promotion behavior scale, it was found that HL significantly and positively predicted nutrition [47,49,52,53].

### 3.6. Quality Assessment of Included Studies

Supplemental Table S4 presents the risk of bias (methodological quality) ratings for the studies included in this systematic review, as assessed using the Newcastle–Ottawa Scale (NOS). All included studies employed a cross-sectional design. In total, 37 studies were assessed, with overall NOS scores ranging from 5 to 10 (median = 8; mean = 7.68). Most studies were rated high quality (n = 31), while six were classified as moderate quality.

Across the NOS domains, selection scores ranged from 3 to 5 (most frequently 4–5), whereas comparability scores showed the most significant variability (0–2; in five studies, this domain received zero points). The outcome domain was rated two points in most studies (and three points in some). These patterns indicate that the main methodological limitations were more often related to control of confounding and comparability than to outcome assessment. In particular, studies that received zero points in the comparability domain did not adequately adjust for key confounders, which may limit the internal validity of their reported associations between HL and health behaviors. Accordingly, findings from these studies should be interpreted with greater caution when considering the overall body of evidence.

## 4. Discussion

This systematic review synthesized evidence on the relationships between adolescents’ HL and key health behaviors. Overall, higher HL was more frequently associated with more favorable behavioral profiles; however, these associations were not uniform across behavioral domains and appeared to depend on how both HL and behaviors were measured, as well as on contextual factors reflected in cross-country differences.

Across the studies, there were 11 measures of HL, and the most often used was the HLSAC. Other, less commonly used, were NVS, HLS-EU-Q16, HLS-EU-47, HELMA, HLAT-8, and AAHL. Some of these instruments were specifically developed to assess adolescents’ HL (i.e., HLSAC, HELMA, and AAHL). Worth mentioning is that in adolescent HL studies, research tools subjectively measure HL. In addition, the use of eleven different HL instruments, most of which rely on self-report and differ in their conceptual coverage (functional vs. multidimensional) and psychometric properties, limits the comparability of findings across studies. While scales such as HLSAC and HELMA demonstrate good content and structural validity, others are narrower or originally validated in adult populations, which may affect their sensitivity to specific adolescent behaviors and contribute to heterogeneity in observed associations. Previous systematic reviews on adolescents’ HL showed some differences. Fleary et al. [21] synthesis of studies showed that the REALM and s-TOFHLA were also used, contrary to our analyzed studies, for HL measures. Thus, studies use various HL assessment instruments, and recently, the most common has been the HLSAC, which has good content and structural validity [74]. Similarly, the validity of HELMA is considered good [74]. It can only be assumed that s-TOFHLA has been used less in recent studies due to poor reliability and validity [74]. Separately, it is worth mentioning HLS19-Q12, which was used in one study [55]. It was developed on the basis of the same conceptual framework and definition of HL as the HLS-EU-Q47 questionnaire and was validated [69] with the 18-and-older population [75]. However, in recent years, it has been successfully validated in the adolescent population [55,76], so it can be expected that it can be more widely applied in further studies. For the digital HL measure, eHEALS and MNH were used, and that replicates the trends observed in previous analyses [21].

From a theoretical perspective, these patterns are consistent with the multidimensional nature of HL, which involves knowledge, motivation, and competencies to access, understand, appraise, and apply health information [8]. Functional skills (e.g., basic reading and numeracy) are likely to be particularly important for tasks such as interpreting labels or simple health messages, whereas interactive and critical competencies are more relevant for appraising the credibility of information, resisting persuasive messaging, and negotiating health-related decisions in social contexts. Multidimensional self-report instruments such as HLSAC, HELMA, HLAT-8, and AAHL are designed to tap into these broader competencies, while functional screening tools like the NVS capture a narrower construct, which may help explain some of the variation in associations observed across behavioral domains.

In the domain of physical activity, most studies reported a significant positive relationship between HL and physical activity. A previous systematic review also found a positive association between HL and physical activity [77]. However, that review included participants of various ages, and only one study focused on adolescents. Another systematic review examined the relationship between adolescents’ HL and health behavior [21], but it included only two studies and used a composite health-promoting behavior score (including physical and sedentary behavior). By synthesizing a larger body of adolescent-focused evidence, our review strengthens the conclusion that HL is meaningfully associated with adolescents’ physical activity. This association may be explained by the fact that individuals with higher HL possess skills that enable them to engage in health-enhancing behaviors such as regular physical activity [78]. Beyond understanding health information, HL empowers individuals to exert greater control over their health and everyday health-related decisions [79].

Despite a documented association between HL and physical activity, we found some mixed results, especially in European settings. This may indicate a stronger moderating role of contextual factors. Opportunities for physical activity depend largely on the school’s physical and social environment and on socioeconomic and built-environment conditions at the community level [80,81]. In such contexts, HL may be necessary but not sufficient for higher activity levels; the behavioral expression of HL may require enabling environments.

Differences in the observed associations also emerged across measurement approaches. In at least one study, significant associations with physical activity were observed when HL was assessed using multidimensional subjective instruments, whereas no association was found when it was measured with a purely functional screening tool. This pattern is consistent with the assumption that broader interactive and critical competencies may be more strongly implicated in health-promoting behaviors than basic functional skills alone. Consistently, when HL was assessed using subjective multidimensional instruments, HL and physical activity were significantly related [58], whereas no relationship emerged when HL was measured using objective functional tools. Notably, similar patterns were reported across different countries, suggesting that measurement choice may partly explain discrepant findings.

Evidence on digital health literacy and physical activity remains scarce, but the available studies point to a potentially increasing role of online information navigation skills. Given the very small number of studies, firm conclusions cannot be drawn; however, these preliminary findings highlight digital HL as a promising direction for future research, particularly in relation to adolescents’ activity opportunities and context-specific constraints.

With regard to substance use-related indicators, the overall pattern was consistent with a protective association, particularly when behavior was assessed as recent practice rather than lifetime experimentation. In studies where no or only minimal adjustment for confounding factors was undertaken, the negative associations between HL and substance use may partly reflect underlying socioeconomic or family influences rather than a direct effect of HL. Such findings should therefore be interpreted as suggestive rather than definitive evidence of a protective role of HL. This distinction is conceptually important: health literacy is likely to be more strongly related to risk appraisal and sustained decision-making [79] than to one-off experimentation that is often socially influenced by peers [82]. Variation in findings across HL instruments further suggests that functional screening tools and broader multidimensional measures capture distinct constructs, which may exhibit different relationships with behavior [78,83]. In the context of substance use, this is plausible, given that the critical and communicative dimensions of HL are conceptually more closely aligned with risk appraisal [56], refusal self-efficacy, and evaluation of persuasive content [84] than with purely functional abilities.

Indicators of nutrition-related behaviors showed the most transparent and consistent associations with HL. A plausible explanation is that food choices are continuously shaped by exposure to information (advertising, labeling, social media content) and everyday decision-making, in which HL—particularly its critical and communicative components—may directly support the appraisal of information quality and resistance to persuasive messaging [85,86]. In cases where weak or no associations were observed, this may be related to measurement specificity (e.g., single-item indicators, differing reference periods) or to behavioral clustering, whereby broader family and school environments constrain choices regardless of individual capabilities.

Methodological considerations

All included studies employed cross-sectional designs, which limit the ability to draw causal conclusions and leave reverse causation as a possibility. In the context of adolescent development, it is plausible that participation in health-promoting activities (e.g., organized sports, school-based health education, or nutrition programs) may itself contribute to the development of HL skills, creating feedback loops rather than a strictly unidirectional ‘HL → behavior’ pathway. Future longitudinal and intervention studies are needed to clarify whether changes in HL precede behavioral change, whether engagement in specific health behaviors enhances HL, or whether both processes operate simultaneously. Substantial heterogeneity in the measurement of both exposure (health literacy) and outcomes (behavioral indicators) also reduces comparability and likely contributes to mixed results in domains such as physical activity and substance use. Furthermore, the risk-of-bias assessment indicated that the most frequent limitations were related to confounder control and group comparability.

It is also worth noting that most included studies originate from Europe, introducing regional bias and limiting generalizability to other communities. It should also be noted that our search was restricted to English language publications, which may have introduced language bias and limited cultural comparability.

We would also like to draw attention to the health-related behavior indicators selected in our study. Our study included health behavior indicators such as physical activity, smoking, alcohol and substance use, and weight-related behaviors, which are identified as the main health behavior indicators in adolescence [87]. However, systematic reviews and meta-analyses also demonstrate that sleep and sleep quality are important for adolescents’ health and well-being [88,89]. The exclusion of this indicator may therefore be considered a limitation of the study. As a systematic review examining the relationship between eHealth literacy and health-related behaviors (including sleep) has already been conducted in adult populations [90], future systematic reviews focusing on adolescent populations should also include this indicator.

Implications for practice and future research

Despite these limitations, the evidence supports a meaningful association between adolescent health literacy and several domains of health behavior, most consistently in nutrition and substance use. From school health and public health perspectives, interventions that strengthen health literacy—particularly critical and digital skills—may help adolescents navigate complex information environments and make healthier choices. Future research should prioritize harmonized measurement of health literacy and behavioral outcomes, transparent reporting of scoring procedures and cut-off values, stronger confounder control based on explicit causal frameworks, and longitudinal and intervention studies that can clarify the directionality of associations and identify those dimensions of health literacy that are most modifiable and most relevant for behavior change.

Although intervention studies are essential for establishing causal relationships, cross-sectional studies remain and will continue to be common in research practice. They are also valuable for identifying associations and enabling cross-cultural comparisons. In our analysis, only two studies included cross-cultural comparisons. Notably, even data from the same region (in our case, Europe) can vary considerably, highlighting the importance of large-scale, multi-country studies. Examples include the European Health Literacy Survey 2019–2021 (M-POHL), which covered 17 European countries (but with adult population) [75], and the Health Behavior in School-aged Children (HBSC) study, conducted among adolescents aged 11, 13, and 15 years across 44 countries and regions in Europe, Central Asia, and Canada. It is worth noting that the health literacy scale was also included in the HBSC protocol. However, comparable large-scale cross-cultural studies focusing on older adolescents are still needed.

## 5. Conclusions

This systematic review analyzed studies on adolescents’ HL and health-related behaviors. The search revealed that a relatively large number of studies have been published in recent years, indicating a growing interest in understanding the importance of HL among adolescents and its relationship with healthy behaviors. Overall, the review found that most recent studies reported a positive association between adolescents’ HL and health-related behaviors. These findings suggest that adolescents with higher HL tend to be more physically active, are less likely to smoke or use alcohol or drugs, and have healthier nutritional behaviors compared with those with lower HL. Furthermore, important gaps in the current research were identified, particularly regarding the use of both subjective and objective measures of HL. Nevertheless, the findings indicate that policies should be developed to support adolescents’ HL development, as the skills required to make informed health decisions are essential during this stage of life.

## Figures and Tables

**Table 1:** Eligibility criteria.

Study inclusion criteria:
The primary population comprised adolescents aged 10–19 years, in line with the WHO definition. Study targeted HL (both general and digital HL) measured by validated research tools. The study targeted specific health behavior(s), including physical activity, alcohol, smoking, substance use, and weight-related behaviors (including diet). The behaviors could be assessed separately or examined as part of a combined measure. The study statistically examined the association between HL and health behavior. The statistical methods employed by the studies were not limited by the heterogeneity in their analysis techniques. The full text was available in English. Studies were published between 1 January 2019 to 31 December 2024.
Study exclusion criteria:
The article type was a case report, review article, systematic analysis, scoping review, study protocol, or qualitative study. The article did not examine associations between HL and health behavior indicators. HL was investigated as an outcome variable rather than as an exposure or explanatory factor. The study focused on other specific forms of HL (e.g., oral health literacy, pregnancy health literacy, sexual and reproductive health literacy, etc.). HL and lifestyle indicators were not measured using validated research instruments. The study sample included both adolescents (under 19 years) and older participants, and results for the subgroup under 19 years were not analyzed or reported separately. The study targeted specific populations such as clinical or patient groups, individuals with overweight, and other specialized populations.

## Data Availability

No new data were created or analyzed in this study. Data sharing is not applicable to this article.

## Supplementary Materials

The following supporting information can be downloaded at https://www.mdpi.com/article/10.3390/epidemiologia7010029/s1, Table S1: Prisma checklist; Table S2: Search strategies used in the systematic review; Table S3: List of selected articles; Table S4: Risk of bias of the studies included in this systematic review, assessed using the Newcastle–Ottawa Scale (NOS).

## Abbreviations

The following abbreviations are used in this manuscript:

HL	Health Literacy
HLSAC	Health Literacy for School-aged Children Scale
HELMA	Health Literacy Measure for Adolescents
HLS-EU-Q16	European Health Literacy Survey Questionnaire
HLS-EU-Q47	European Health Literacy Survey Questionnaire
AAHL	Assessments of Adolescent Health Literacy
NVS	The Newest Vital Sign
HLS-47	47-item Health Literacy Study-Asia-Questionnaire
HLAT-8	The Eight-Item Health Literacy Assessment Tool
eHEALS	e-Health Literacy
CAIHLQ	The Chinese Adolescent Interactive Health Literacy Questionnaire
MHL	Media Health Literacy
BMI	Body Mass Index
