# Supplementary material for: Systematic Review of Health Literacy and Health Behavior in Adolescents Research
Source: Epidemiologia (Basel). 2026 Feb 18;7(1):29. doi: 10.3390/epidemiologia7010029 (PMC12939913; doi:10.3390/epidemiologia7010029)
Supplement: Supplementary file 1 [file epidemiologia-07-00029-s001.zip › Suplemental Table S3_Selected_articles.pdf]

Suplemental Table S3 List of selected articles

1. Ayaz-Alkaya, S.; Kulakçı-Altıntaş, H. Nutrition-exercise behaviors, health literacy level, and related factors in adolescents in turkey. *J. Sch. Health* **2021**, *91*, 625–631. doi:10.1111/josh.13057
2. Ayaz-Alkaya, S.; Kulakçi-Altintaş, H. Predisposing factors of health promotion behaviors and health literacy in adolescents: A cross-sectional design. *PNH*. **2024**, *41*, 416–422. doi:10.1111/phn.13294
3. Azarang, Z.; Farahaninia, M.; Bozorgnezhad, M.; Haghani, H. Relationship Between Health Literacy and Addiction Susceptibility in High School Adolescents. *J. Client-Centered Nurs. Care* **2024**, *10*, 3-14. doi:10.32598/JCCNC.10.1.93.21
4. Bektas, İ.; Kudubeş, A.A.; Ayar, D.; Bektas, M. Predicting the healthy lifestyle behaviors of Turkish adolescents based on their health literacy and self-efficacy levels. *J. Pediatr. Nurs*. **2021**, *59*, e20–e25. doi:10.1016/j.pedn.2021.01.016
5. Brandt, L.; Schultes, M.; Yanagida, T.; Maier, G.; Kollmayer, M.; Spiel, C. Differential associations of health literacy with Austrian adolescents' tobacco and alcohol use. *Public Health* **2019**, *174*, 74–82. doi:10.1016/j.puhe.2019.05.033
6. Delbosq, S.; Velasco, V.; Vercesi, C.; Vecchio, L. P. Adolescents' nutrition: The role of health literacy, family and socio-demographic variables. *Int. J. Environ. Res. Public Health* **2022**, *19*. doi:10.3390/ijerph192315719
7. Duplaga, M.; Grysztar, M. Nutritional behaviors, health literacy, and health locus of control of secondary schoolers in southern poland: A cross-sectional study. *Nutrients* **2021**, *13*, 4323. doi:10.3390/nu13124323
8. Duplaga, M.; Grysztar, M. The use of e-cigarettes among high school students in Poland is associated with health locus of control but not with health literacy: A cross-sectional study. *Toxics* **2022,** *10*, 41. doi:10.3390/toxics10010041
9. Fleary, S. A. The relationship between health literacy and correlates of adolescents' obesogenic and substance use behaviors. *J. Pediatr. Nurs*. **2023**, *70*, e40–e47. doi:10.1016/j.pedn.2022.11.030
10. Fleary, S.A.; Joseph, P.L. Health literacy and health behaviors in parent-adolescent dyads: An actor-partner interdependence model approach. *Psychol. Health* **2024**, *39*, 803–822. doi:10.1080/08870446.2022.2117809
11. Fleary, S.A.; Rastogi, S.; Srivastava, V. Adolescent health literacy: Sociodemographic determinants and its relationship with substance use avoidance. *Health Promot. Int*. **2024**, *39*, daae194. doi:10.1093/heapro/daae194.
12. Guo, S.; Yu, X.; Davis, E.; Armstrong, R.; Riggs, E.; Naccarella, L. Adolescent health literacy in Beijing and Melbourne: A cross-cultural comparison. *Int. J. Environ. Res. Public Health* **2020,** *17* doi:10.3390/ijerph17041242
13. Guo, S.; Naccarella, L.; Yu, X.; Armstrong, R.; Browne, G.; Shi, Y.; Davis, E. Health literacy and its mediating role in predicting health behaviors among Chinese secondary students. *APJPH* 2021, *33*, 76–83.
14. Gürkan, K.P.; Ayar, D. The impact of e-health literacy on health promotion behaviors of high school students. *J. Pediatr. Res*. **2020**, *7*, 286–292. doi:10.4274/JPR.GALENOS.2019.81488
15. Hnidková, L.; Bakalár, P.; Magda, R.; Kolarčik, P.; Kopčáková, J.; Boberová, Z. Adolescents' health literacy is directly associated with their physical activity but indirectly with their body composition and cardiorespiratory fitness: Mediation analysis of the slovak HBSC study data. *BMC Public Health* **2024**, *24* doi:10.1186/s12889-024-20227-z
16. Huang, J.; Chan, S.C.; Keung, V.M.W.; Cheung, C.K.M.; Lo, A.S.C.; Lau, V.T.C.; Mui, L.W.H.; Lee, A.; Wong, M.C.S. Associations between GoSmart channel, health literacy and health behaviours in adolescents: A population-based study. *Health Expect*. **2024**, *27*, e13894. doi: 10.1111/hex.13894.
17. Jindarattanaporn, N.; Rittirong, J.; Phulkerd, S.; Thapsuwan, S.; Thongcharoenchupong, N. Are exposure to health information and media health literacy associated with fruit and vegetable consumption? *BMC Public Health* **2023**, *23*, 1554. doi:10.1186/s12889-023-16474-1
18. Kanellopoulou, A.; Notara, V.; Antonogeorgos, G.; Chrissini, M.; Rojas-Gil, A.; Kornilaki, E.N.; Lagiou, A.; Panagiotakos, D.B. Inverse association between health literacy and obesity among children in greece: A school-based, cross-sectional epidemiological study. *Health Educ Behav*. **2022**, *49*, 54-65. doi:10.1177/1090198120982944
19. Karagözoğlu, M.; İlhan, N. The effect of health literacy on health behaviors in a sample of turkish adolescents. *J. Pediatr. Nurs*. **2024**, *77*, e187–e194. doi:10.1016/j.pedn.2024.04.028
20. Kesic, M.G.; Savicevic, A.J.; Peric, M.; Gilic, B.; Zenic, N. Specificity of the associations between indices of cardiovascular health with health literacy and physical literacy; A cross-sectional study in older adolescents. *Medicina*, **2022**, *58*, 1316. doi:10.3390/medicina58101316
21. Kinnunen, J.M.; Paakkari, L.; Rimpelä, A.H.; Kulmala, M.; Richter, M.; Kuipers, M.A.G.; .Kunst, A.E.; Lindfors, P.L. The role of health literacy in the association between academic performance and substance use. *Eur. J. Public Health* **2022**, *32*, 182–187. doi:10.1093/eurpub/ckab213
22. Kleszczewska, D.; Mazur, J.; Porwit, K.; Kowalewska, A. Who is able to resist what is forbidden? - the relationship between health literacy and risk behaviours in secondary school students in the broader social and educational context. *Int. J. Environ. Res. Public Health* **2022**, *19*, 9381. doi:10.3390/ijerph19159381

Table S3 (continue)

23. Korkmaz Aslan, G.; Kartal, A.; Turan, T.; Taşdemir Yiğitoğlu, G.; Kocakabak, C. Association of electronic health literacy with health-promoting behaviours in adolescents. *Int. J. Nurs. Pract*. **2021**, *27*, e12921. doi:10.1111/ijn.12921
24. McCormick, B.A.; Porter, K.J.; You, W.; Yuhas, M.; Reid, A.L.; Thatcher, E.J.; Zoellner, J.M. Applying the socio-ecological model to understand factors associated with sugar-sweetened beverage behaviours among rural Appalachian adolescents. *Public Health Nutr*. **2021**, *24*, 3242–3252. doi:10.1017/S1368980021000069
25. Motemedi, M.; Peyman, N.; Afzalaghaee, M. Relationship of health literacy and regular physical activity self-efficacy with body mass index in adolescent girls aged 15-18 years. *J. Health Lit*. **2020**, *5*, 64–73.
26. Ozturk Eyimaya, A.; Tezel, A. Evaluation of adolescents' cardiovascular health behaviors and health literacy levels in turkey. *J. Pediatr. Nurs*. **2024**, *79*, e24–e30. doi:10.1016/j.pedn.2024.09.014
27. Ozturk Haney, M. Health literacy and predictors of body weight in Turkish children. *J. Pediatr. Nurs*. **2020**, *55*, e257–e262. doi:10.1016/j.pedn.2020.05.012
28. Ozturk, F.O.; Ayaz-Alkaya, S. Health literacy and health promotion behaviors of adolescents in turkey. *J. Pediatr. Nurs.* **2020**, *54*, e31–e35. doi:10.1016/j.pedn.2020.04.019
29. Paakkari, L.T.; Torppa, M.P.; Paakkari, O.-P.; Välimaa, R.S.; Ojala, K.S.A.; Tynjälä, J.A. Does health literacy explain the link between structural stratifiers and adolescent health? *Eur. J. Public Health* **2019**, *29*, 919–924. doi:10.1093/eurpub/ckz011
30. Prihanto, J.B.; Nurhayati, F.; Wahjuni, E.S.; Matsuyama, R.; Tsunematsu, M.; Kakehashi, M. Health literacy and health behavior: Associated factors in Surabaya high school students, Indonesia. *Int. J. Environ. Res. Public Health* **2021**, *18*, 8111. doi:10.3390/ijerph18158111
31. Puupponen, M.; Tynjälä, J.; Tolvanen, A.; Välimaa, R.; Paakkari, L. Energy drink consumption among Finnish adolescents: Prevalence, associated background factors, individual resources, and family factors. Int. *J. Public Health* **2021**, *7*, 620268. doi:10.3389/ijph.2021.620268
32. Reid, A.L.; Porter, K.J.; You, W.; Kirkpatrick, B.M.; Yuhas, M.; Vaught, S.S.; Zoellner, J.M. Low health literacy is associated with energy-balance-related behaviors, quality of life, and BMI among rural Appalachian middle school students: A cross-sectional study. *J. Sch. Health* **2021**, *91*, 608–616. doi:10.1111/josh.13051
33. Sukys, S.; Kuzmarskiene, G.; Motiejunaite, K. Exploring the association between adolescents' health literacy and health behavior by using the short health literacy (HLS(19)-Q12) questionnaire. *Healthcare* **2024**, *12*, 2585. doi:10.3390/healthcare12242585
34. Sukys, S.; Tilindiene, I.; Trinkuniene, L. Association between health literacy and leisure time physical activity among lithuanian adolescents. *Health and Social Care in the Community*, **2021**, *29*, e387–e395. doi:10.1111/hsc.13363
35. Rutkauskaite, R.; Kuusinen, K. Links between adolescents’ health literacy and their physical activity and body mass index. *Balt. J. Sports Health Sci.* **2019**, *3*, 4–14. https://doi.org/10.33607/bjshs
36. Yang, R.; Li, D.; Hu, J.; Tian, R.; Wan, Y.; Tao, F.; Fang, J.; Zhang, S. Association between health literacy and subgroups of health risk behaviors among Chinese adolescents in six cities: A study using regression mixture modeling. *Int. J. Environ. Res. Public Health* **2019**, *16*, 3680. doi:10.3390/ijerph16193680. doi:10.3390/ijerph16193680
37. Zare-Zardiny, M.R.; Abazari, F.; Zakeri, M.A.; Dastras, M.; Farokhzadian, J. The association between body mass index and health literacy in high school students: A cross-sectional study. *J. Educ. Health Promot.* **2021**, *10*, 431. doi:10.4103/jehp.jehp_96_21.
