# Supplementary material for: Systematic Review of Health Literacy and Health Behavior in Adolescents Research
Source: Epidemiologia (Basel). 2026 Feb 18;7(1):29. doi: 10.3390/epidemiologia7010029 (PMC12939913; doi:10.3390/epidemiologia7010029)
Supplement: Supplementary file 1 [file epidemiologia-07-00029-s001.zip › Suplemental Table S4_bias assessment.pdf]

Suplemental Table S4. Risk of bias of the studies included in this systematic review, assessed using the Newcastle–Ottawa Scale (NOS)

Author(s), (year)	Study Design	Selection	Comparability	Outcome	Total Score	Quality Rating
Ayaz-Alkaya et al., 2021	cross-sectional	3	0	2	5	Moderate
Ayaz-Alkaya et al., 2024	cross-sectional	3	2	2	7	High
Azarang et al., 2024	cross-sectional	4	2	2	8	High
Bektas et al. 2021	cross-sectional	3	2	2	7	High
Brand et al. 2019	cross-sectional	3	1	2	6	Moderate
Delbosq et al. 2022	cross-sectional	3	2	2	7	High
Duplaga et al. 2021	cross-sectional	3	2	2	7	High
Duplaga et al., 2022	cross-sectional	3	2	2	7	High
Fleary et al. 2023	cross-sectional	4	2	2	8	High
Fleary & Joseph 2024	cross-sectional	4	2	2	8	High
Fleary et al., 2024	cross-sectional	4	2	2	8	High
Guo et al., 2020	cross-sectional	4	2	2	8	High
Guo et al., 2021	cross-sectional	5	1	2	8	High
Gürkan, & Ayar, 2020	cross-sectional	4	0	2	6	Moderate
Hnidkova et al., 2024	cross-sectional	5	2	3	10	High
Huang et al., 2024	cross-sectional	4	1	2	7	High
Jindarattanaporn et al., 2023	cross-sectional	5	2	2	9	High
Kanellopoulou et al., 2022	cross-sectional	5	2	3	10	High
Karagözoğlu & İlhan, 2024	cross-sectional	4	1	2	7	High
Kesic et al., 2022	cross-sectional	5	1	3	9	High
Kinnunen et al., 2022	cross-sectional	5	2	2	9	High
Kleszczewska et al., 2022	cross-sectional	5	2	2	9	High
Korkmaz Aslan et al., 2021	cross-sectional	5	1	2	8	High
McCormick et al., 2021	cross-sectional	3	2	2	7	High
Motemedi et al., 2020	cross-sectional	3	1	2	6	Moderate
Ozturk Eyimaya & Tezel, 2024	cross-sectional	4	1	2	7	High
Ozturk Haney, 2020	cross-sectional	3	2	3	8	High
Ozturk & Ayaz-Alkaya, 2020	cross-sectional	5	0	2	7	High
Paakkari et al., 2019	cross-sectional	5	2	2	9	High
Prihanto et al., 2021	cross-sectional	3	2	2	7	High
Puupponen et al., 2021	cross-sectional	5	2	2	9	High
Reid et al., 2021	cross-sectional	3	0	3	6	Moderate
Sukys et al., 2024	cross-sectional	4	2	2	8	High
Sukys et al., 2021	cross-sectional	5	2	2	9	High
Rutkauskaite & Kuusinen, 2019	cross-sectional	3	0	2	5	Moderate
Yang et al., 2019	cross-sectional	5	2	2	9	High
Zare-Zardiny et al., 2021	cross-sectional	5	2	2	9	High
