# Supplementary material for: Systematic Review of Health Literacy and Health Behavior in Adolescents Research
Source: Epidemiologia (Basel). 2026 Feb 18;7(1):29. doi: 10.3390/epidemiologia7010029 (PMC12939913; doi:10.3390/epidemiologia7010029)
Supplement: Supplementary file 1 [file epidemiologia-07-00029-s001.zip › Supplemental Table S2_Search_strategies.pdf]

Supplemental Table S2. Search strategies used in the systematic review.

(("adolescen*"[Title/Abstract] OR "teen*"[Title/Abstract] OR schoolchildren[Title/Abstract] OR youth[Title/Abstract]) AND (health literacy[Title/Abstract] OR digital health literacy[Title/Abstract] OR media health literacy[Title/Abstract]) AND ("health* behavio*r"[Title/Abstract] OR health[Title/Abstract] OR physical activity[Title/Abstract] OR "exercise*"[Title/Abstract] OR alcohol[Title/Abstract] OR drinking[Title/Abstract] OR smoking[Title/Abstract] OR tobacco use[Title/Abstract] OR body weight[Title/Abstract] OR nutrition[Title/Abstract] OR diet[Title/Abstract]))	PubMed
TITLE-ABS-KEY (("adolescen*" OR "teen*" OR "schoolchildren" OR "youth")) AND TITLE-ABS-KEY (("health literacy" OR "digital health literacy" OR "media health literacy ")) AND TITLE-ABS-KEY (("health* behavio*r " OR "health" OR "physical activity" OR "exercise*" OR "alcohol" OR "drinking" OR "smoking" OR "tobacco use" OR "body weight" OR "nutrition" OR "diet")) AND PUBYEAR > 2018 AND PUBYEAR < 2025 AND (LIMIT-TO (DOCTYPE , "ar"))	Scopus
("adolescen*" OR "teen*" OR schoolchildren OR youth) AND ("health literacy" OR "digital health literacy" OR "media health literacy") AND ("health* behavio*r" OR health OR "physical activity" OR "exercise*" OR alcohol OR drinking OR smoking OR "tobacco use" OR "body weight" OR nutrition OR diet)<br>Title<br>("adolescen*" OR "teen*" OR schoolchildren OR youth) AND ("health literacy" OR "digital health literacy" OR "media health literacy") AND ("health* behavio*r" OR health OR "physical activity" OR "exercise*" OR alcohol OR drinking OR smoking OR "tobacco use" OR "body weight" OR nutrition OR diet)<br>Abstract<br>("adolescen*" OR "teen*" OR schoolchildren OR youth) AND ("health literacy" OR "digital health literacy" OR "media health literacy") AND ("health* behavio*r" OR health OR "physical activity" OR "exercise*" OR alcohol OR drinking OR smoking OR "tobacco use" OR "body weight" OR nutrition OR diet)<br>Keywors	Psychinfo
